# Spontaneous Bacterial Peritonitis in Advanced Cirrhosis: Diagnosis by Tm Mapping and Inflammatory Profiles of Extracellular Vesicles

**DOI:** 10.3390/jcm14145096

**Published:** 2025-07-17

**Authors:** Aiko Murayama, Kazuto Tajiri, Nozomu Muraishi, Yuka Hayashi, Masami Minemura, Hideki Niimi, Ichiro Yasuda

**Affiliations:** 1 Third Department of Internal Medicine, Faculty of Medicine, Academic Assembly, University of Toyama, 2630 Sugitani, Toyama 930-0194, Japan; aikokikuchi@me.com (A.M.); yasudaic@med.u-toyama.ac.jp (I.Y.); 2 Department of Clinical Laboratory and Molecular Pathology, Graduate School of Medicine and Pharmaceutical Science for Research, University of Toyama, 2630 Sugitani, Toyama 930-1094, Japan

**Keywords:** cirrhosis, extracellular vesicles, macrophages, spontaneous bacterial peritonitis

## Abstract

**Background/Objectives**: Ascites is a major complication in patients with decompensated cirrhosis. Spontaneous bacterial peritonitis (SBP), an infection of the ascitic fluid, is a life-threatening condition in patients with cirrhosis. This study aimed to assess the utility of Tm mapping, a novel high-efficacy method for bacterial detection and quantification, in the early diagnosis of SBP and its pathogenesis. **Methods**: Ascitic fluid samples from 29 patients with cirrhosis were analyzed using Tm mapping for bacterial identification. Inflammatory cytokine and pathogen-associated molecular pattern levels in ascitic fluid were measured and correlated with SBP pathophysiology. Additionally, the role of ascitic macrophages was investigated in vitro. **Results**: Tm mapping detected bacteria more effectively than conventional culture methods. In samples where bacteria were identified, ascitic interleukin (IL)-6 levels were elevated. A positive correlation was observed between extracellular vesicle (EV) levels and IL-6, suggesting a role for EVs in peritoneal inflammation. Furthermore, EVs derived from Gram-negative bacteria induced M1 macrophage differentiation via the signal transducer and activator of transcription 1 signaling pathway. **Conclusions**: Tm mapping is a valuable tool for the early detection of bacteria in ascitic fluid. Additionally, EVs promote M1 macrophage differentiation, implicating them in the pathogenesis of cirrhotic complications, including SBP.

## 1. Introduction

In patients with cirrhosis, ascites frequently develops as a result of hypoalbuminemia and portal hypertension [1]. Ascites not only impairs quality of life but also leads to complications such as infections, electrolyte disturbances, and hepatorenal syndrome. Spontaneous bacterial peritonitis (SBP) is one of the most common infections in decompensated patients with cirrhosis who have ascites, occurring in 10–30% of hospitalized patients with cirrhosis [2,3]. The prognosis for SBP is poor, with an estimated 1-year survival of approximately 40% following its onset, primarily due to the development of acute kidney injury and acute exacerbation of chronic liver failure [2,4,5]. Therefore, early diagnosis of SBP is vital for improving the prognosis of decompensated patients with cirrhosis.

SBP is diagnosed when an ascitic fluid culture is positive or when the neutrophil count in ascitic fluid exceeds 250 /μL [3,6,7]. However, the positivity rate of ascitic fluid cultures is relatively low, ranging from 42% to 65%, even in cases with elevated neutrophil counts [2,3,7,8]. Furthermore, because culture results require time for incubation, empirical antibiotic therapy is often initiated. Recently, hospital-acquired SBP and infections with multidrug-resistant bacteria have become more common, reducing the effectiveness of third-generation cephalosporins, which are typically used as the first-line empirical therapy [1,2,3,4,6,7,9,10]. Therefore, a more sensitive and rapid diagnostic method is needed for SBP.

Niimi et al. developed a novel rapid bacterial identification system for sepsis [11]. This system directly extracts DNA from patient samples, followed by nested PCR using seven universal bacterial primers targeting conserved regions of the 16S ribosomal RNA gene. The melting temperatures (Tm) of the seven PCR products are then mapped in two dimensions and compared with a database to rapidly identify the bacteria [11]. Simultaneously, a control line using *Escherichia coli* (*E. coli*) is included for bacterial quantification. Using this method, named Tm mapping, bacterial identification and quantification can be completed within 3 h [11,12]. Tm mapping has demonstrated utility in previous studies involving various clinical samples, including blood, intraocular fluid, amniotic fluid, and pus [11,13,14,15,16]. In this study, we aim to evaluate the utility of the Tm mapping method for diagnosing SBP using ascitic fluid samples.

The pathophysiology of SBP is thought to be influenced by several factors, such as abnormal gut microbiota proliferation, immune dysfunction, and increased intestinal permeability, the phenomenon known as “leaky gut” that facilitates bacterial translocation [4,10,17,18]. Impaired intestinal barrier function allows not only the migration of bacteria but also the release of pathogen-associated molecular patterns (PAMPs) from pathogens, which stimulate and activate macrophages [18,19,20]. However, the role of PAMPs in SBP pathogenesis remains unclear. On the other hand, extracellular vesicles (EVs), which are lipid bilayer-enclosed particles released by virtually all types of cells, have recently attracted considerable attention in biomedical research. EVs derived from Gram-negative bacteria contain lipopolysaccharide (LPS), a component of the bacterial cell wall and a well-known PAMP [21]. These vesicles are capable of transporting various molecules, including proteins and mRNA, thereby mediating intercellular communication [21,22]. In clinical practice, we frequently encounter cases in which ascitic fluid cultures are negative despite an elevated white blood cell (WBC) count. This suggests that in patients with advanced cirrhosis with ascites, disease-promoting substances derived from bacteria may translocate before bacterial migration from the intestine to the ascitic fluid occurs. To investigate this, we examined the relationship between cytokines and PAMPs, including EVs, in ascitic fluid and SBP pathogenesis, as well as the dynamics of ascitic macrophages.

## 2. Materials and Methods

### 2.1. Study Design and Subjects

This was an observational study conducted at a single facility (Toyama University Hospital, Toyama, Japan). Patients with cirrhosis who presented with progressive ascites between October 2019 and October 2022 were enrolled. The diagnosis of cirrhosis was made by expert hepatologists with over 15 years of experience and was based on a comprehensive assessment of blood tests and imaging studies, including abdominal ultrasounds and CT scans. Written informed consent for the collection and use of ascitic fluid samples was obtained from all participants. The study was approved by the Ethical Review Committee of Toyama University (Approval No. R2020092).

Ascitic fluid samples were obtained via paracentesis. The timing of fluid collection was determined by the attending physician based on clinical suspicion of infection or the detection of increasing ascites. At the time of sample collection, routine clinical tests were performed, including biochemical analyses, cell counts, and ascitic fluid cultures. Background clinical data were obtained from electronic medical records. SBP was diagnosed based on neutrophil counts > 250 /µL or positive bacterial cultures in ascitic fluid [2,3,6,7]. Since neutrophil counts in ascitic fluid could not be measured at our facility, WBC ≥ 350 /µL was deemed a surrogate ascitic neutrophil count of 250 /µL.

### 2.2. Tm Mapping

Ascitic fluid samples were also analyzed using Tm mapping, as previously described [11]. Briefly, the difference from the mean value of each of the seven Tm values was calculated, and the total difference value (DV) from the database was obtained. The Tm mapping shape difference was represented by the DV. The microorganism was identified as the species with the DV closest to zero, but if the DV exceeded 0.5, specificity could not be determined, and bacterial presence was reported without identifying the pathogen. The quantitative results of the bacterial count were initially calculated by converting them into the bacterial count of *E. coli*, which was the quantitative control. Then, the bacterial concentration was corrected according to the 16S ribosomal RNA operon copy number of the pathogen identified by Tm mapping. If the pathogen was not identified by Tm mapping, the quantitative result was reported as an *E. coli* equivalent. Cases with bacterial signals detected by Tm mapping were deemed Tm mapping-positive, and those with no detectable response as Tm mapping-negative.

### 2.3. ELISA

Cytokine and PAMP concentrations in ascitic fluid were measured using enzyme-linked immunosorbent assay (ELISA). Ascitic fluid samples stored at −80 °C were thawed and analyzed using commercially available ELISA kits according to the manufacturers’ instructions. The ELISA kits used were as follows: interleukin (IL)-6 (R&D Systems, Minneapolis, MN, USA), IL-8 (R&D Systems), LPS (Cloud-Clone, Katy, TX, USA), and EVs (Cosmo Bio, Tokyo, Japan).

### 2.4. Flow Cytometry

Macrophage differentiation in ascitic fluid and in vitro models was analyzed using flow cytometry. FITC-conjugated CD11b antibody (BD Pharmingen, San Diego, CA, USA), PE-conjugated CD80 antibody (BioLegend, San Diego, CA, USA), and PE-conjugated CD163 antibody (eBioscience Inc., San Diego, CA, USA) were used for the analyses. After staining, cells were washed with phosphate-buffered saline, and 500 µL of a paraformaldehyde fixative (Flow Fix, 2% Paraformaldehyde Fixative Kit, Polysciences, Inc., Tokyo, Japan) was added. Fluorescence was measured using a FACS Canto II (BD Biosciences, San Jose, CA, USA). In this study, M1 macrophages were defined as CD11b+/CD80+ cells, while M2 macrophages were defined as CD11b+/CD163+ cells. The M1/M2 ratio was calculated as the percentage of M1 macrophages divided by the percentage of M2 macrophages.

### 2.5. Cell Cultures

Human monocyte-derived THP-1 cells (ATCC, Manassas, VA, USA) were cultured in an RPMI-1640 medium supplemented with 10% fetal bovine serum at 37 °C with 5% CO_2_. Differentiation into M0 macrophages was induced by adding 5 ng/mL of phorbol 12-myristate 13-acetate (Wako, Osaka, Japan). The resulting M0 macrophages were stimulated with 10 ng/mL of LPS (Novus biologicals, LLC, Centennial, CO, USA) and 10 ng/mL of interferon (IFN)-γ (Wako) to induce M1 differentiation. Differentiated M0 macrophages were stimulated with EVs derived from *E. coli* DH5α (Cosmo Bio, Tokyo, Japan) to investigate macrophage dynamics.

### 2.6. Western Blot for Detection of Macrophage Activation

Western blotting was performed to analyze the activation pathway of macrophages. THP-1 cells were harvested 2 h after stimulation, lysed in lysis buffer, and incubated on ice for 30 min. The lysate was then centrifuged at 14,000× *g* for 10 min at 4 °C to extract proteins. A 3 µg amount of protein per sample was loaded onto 4–20% Mini-PROTEAN TGX Precast Gels (Bio-Rad, Hercules, CA, USA) and subjected to electrophoresis using a PowerPac system (Bio-Rad). Following electrophoresis, proteins were transferred onto an Immobilon-P membrane (Merck, Rahway, NJ, USA) and blocked with 4% BlockAce solution (Yukijirushi, Sapporo, Japan). For immunodetection, signal transducer and activator of transcription 1 (STAT1) monoclonal antibody (1:10,000, Proteintech, Rosemont, IL, USA) and phospho-STAT1 (Tyr701) and recombinant antibody (1:10,000, Proteintech) were used as primary antibodies. Horseradish peroxidase-conjugated anti-mouse IgG or anti-rabbit IgG (Agilent Technologies, Yishun Ave, Singapore) were used as secondary antibodies, as appropriate. Protein expression was visualized using an ImageQuant LAS 4000 system (GE Healthcare, Chicago, IL, USA).

### 2.7. Statistical Analysis

Data were presented as medians or means with ranges. Comparisons between groups were performed using the Mann–Whitney U test. To assess the relationship between two variables, linear regression analysis was conducted, and a regression model was fitted to the data. The coefficient of determination (R^2^) and the *p*-values were calculated for evaluation. Statistical analysis was performed using JMP Pro 17.0.0, with *p* < 0.05 deemed statistically significant.

## 3. Results

### 3.1. Patients’ Characteristics

The 29 patients included had decompensated cirrhosis and ascites. Their clinical characteristics are summarized in Table 1. The median age was 69 years (range: 48–94), and 17 patients (58.6%) were male. The majority (24 patients, 82.8%) had non-viral liver disease as the underlying etiology. The median Child–Pugh score was 10 (range: 8–14), with 21 patients (72.4%) classified as Child–Pugh grade C. The median MELD-Na score (Model for End-Stage Liver Disease Sodium score), a validated measure of cirrhosis severity [23], was 10.1 (range: 0.8–23.8). The median Fib-4 index, a marker of liver fibrosis [24,25], was 6.83 (range: 1.46–41.51), indicating advanced fibrosis. Regarding complications, 12 patients (41.4%) had esophageal varices, and 8 (27.6%) had hepatocellular carcinoma. Many patients exhibited symptoms suggestive of SBP, including fever (14 patients, 48.3%), abdominal pain (24 patients, 82.8%), diarrhea (23 patients, 79.3%), and nausea or vomiting (27 patients, 93.1%). Blood tests revealed elevated peripheral WBC count (median: 5940 /µL, range: 2640–21,900) and C-reactive protein (median: 1.58 mg/dL, range: 0.14–18.9), indicating systemic inflammation. Thrombocytopenia (median platelet count: 8.5 × 10^4^/μL, range: 1.5–22.6) and hypoalbuminemia (median: 2.2 g/dL, range: 1.0–3.2) were also observed. Ascitic fluid analysis showed a median WBC count of 330 /µL (range: 30–13,740). The median ascitic albumin level was 0.6 g/dL (range: 0.1–1.8), and the median serum ascites albumin gradient was 1.6 g/dL (range: 0.8–2.4). At the time of analysis, 10 patients (34.5%) had received antibiotics.

### 3.2. Diagnosis of SBP

**Figure 1:**
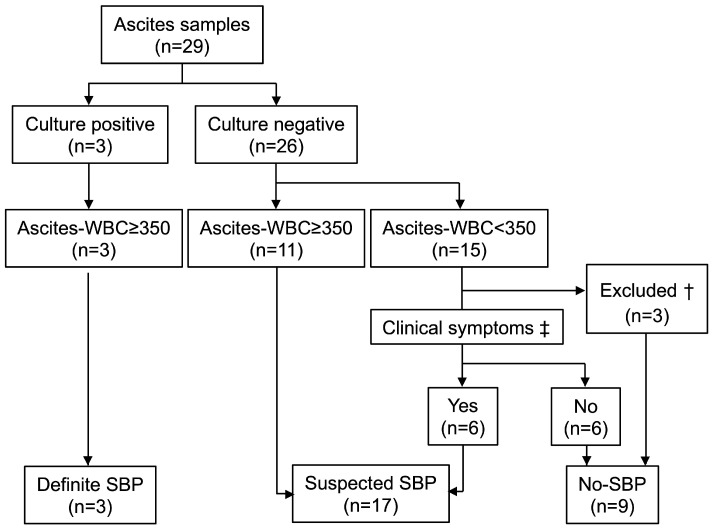
Diagnosis of spontaneous bacterial peritonitis. Patients were classified based on ascitic culture results, ascitic WBC count, and the presence of clinical symptoms. † Cases with sources of infection outside the abdominal cavity were excluded. ‡ Clinical symptoms included fever, abdominal pain, diarrhea, and nausea/vomiting. Ascites-WBC: ascitic white blood cell count.

Based on ascitic fluid analysis and clinical manifestations, patients were categorized into three groups (Figure 1). Ascitic fluid cultures identified causative bacteria in 3 of 29 cases (*Enterococcus faecium* in 1 case, and *Klebsiella pneumoniae* in 2 cases). In these cases, the ascitic WBC count was ≥350 /µL, meeting the diagnostic criteria for definite SBP. Among the remaining cases, 11 patients had an ascitic WBC count of ≥350 /µL despite negative cultures, and 6 had an ascitic WBC count of <350 /µL but presented with clinical symptoms such as fever, abdominal pain, or diarrhea. Although they did not meet the conventional SBP criteria, they were categorized as suspected SBP (*n* = 17). For the remaining nine patients, six had an ascitic WBC count of <350 /µL with no clinical symptoms, and three had an identified extra-abdominal source of infection (cellulitis, suppurative knee arthritis, and pulmonary abscess). They were deemed no-SBP, as no ascitic infection was suspected.

### 3.3. Utility of Tm Mapping for Diagnosing SBP

Using Tm mapping, we identified the same bacterium in cases of definite SBP as detected by conventional culture (Table 2). However, when multiple bacterial species were present, Tm mapping was unable to distinguish them. In cases of suspected SBP (where no bacteria were identified using conventional culture), Tm mapping was able to detect bacterial species in some instances or indicate the presence of multiple organisms (denoted as “Multiple”). Even in cases where bacterial identification was not achieved (denoted as “Unknown”), Tm mapping detected a response to small amounts of bacteria. In those cases, an elevated ascitic WBC count was also observed.

### 3.4. Correlation Between Cytokines in Ascitic Fluid and Clinical Data

**Figure 2:**
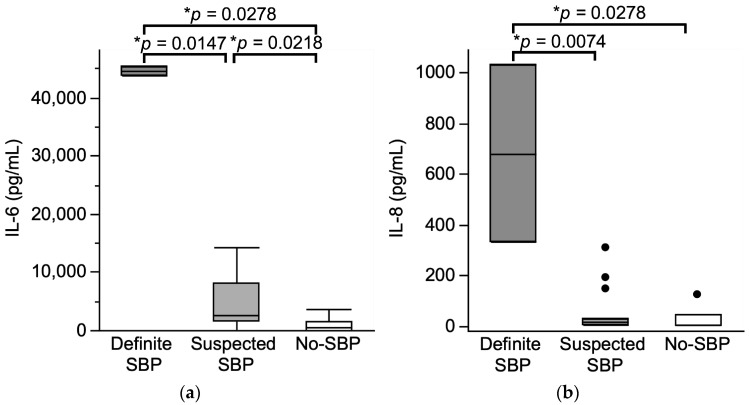

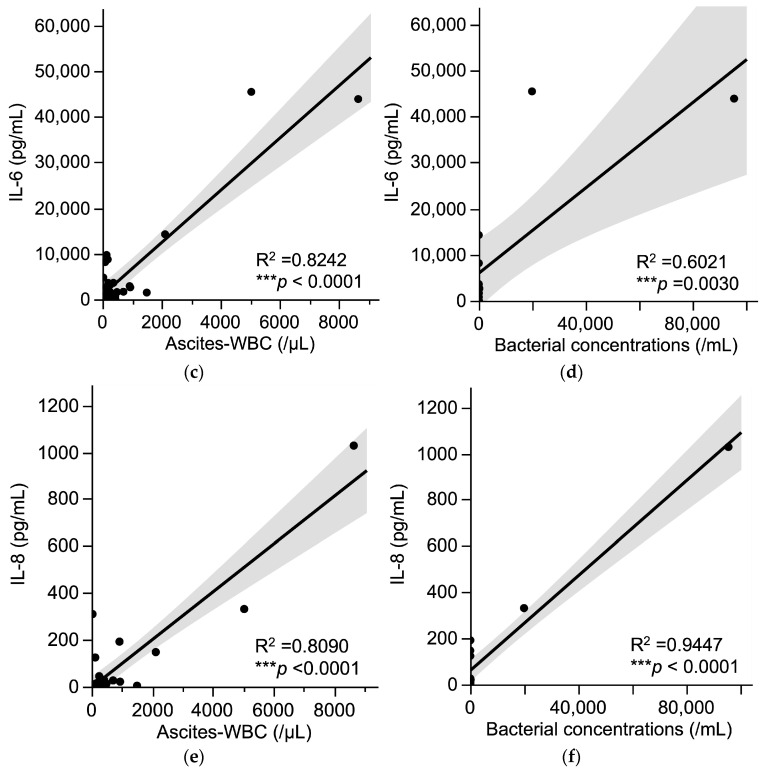
Analysis of cytokines in ascitic fluid. Correlation between SBP diagnosis and cytokine levels in ascitic fluid (**a**,**b**) and correlation between cytokine levels in ascitic test results (**c**–**f**). (**a**) Correlation between SBP diagnosis and IL-6 levels in ascitic fluid. Mean values with 95% confidence intervals for each group: definite SBP group: 44,657.1 (CI: 3467.6–54,642.5), suspected SBP group: 4215.0 (CI: 1934.2–6495.9), and no SBP group: 1008.9 (CI: –195.1 to 2212.8). (**b**) Correlation between SBP diagnosis and IL-8 levels in ascitic fluid. Mean values with 95% confidence intervals: definite SBP group: 679.5 (CI: –3747.3 to 5106.3), suspected SBP group: 53.2 (CI: 3.1–103.3), and no SBP group: 26.8 (CI: –15.3 to 68.8). * *p* < 0.05, Mann–Whitney U test. (**c**,**d**) Correlation between IL-6 levels in ascitic fluid and ascitic test results. (**e**,**f**) Correlation between IL-8 levels in ascitic fluid and ascitic test results. The X-axis represents ascitic test results (ascitic WBC count and bacterial levels measured by the Tm mapping method). The Y-axis represents cytokine levels (IL-6 and IL-8) measured by ELISA. Scatter plots include regression lines with the shaded area indicating the 95% confidence interval. Regression analysis was performed to assess relationships between variables with the coefficient of determination (R^2^) and *p*-value (*** *p* < 0.001). Ascites-WBC: ascitic white blood cell count.

To investigate the pathophysiology of SBP, we analyzed cytokines (IL-6, IL-8) in ascitic fluid and examined their correlation with SBP status (Figure 2). For IL-6, significant differences were observed between definite- and suspected-SBP (median: 44,657.8 pg/mL vs. 2647.4 pg/mL; *p* = 0.0147), as well as between suspected- and no-SBP (median: 2647.4 pg/mL vs. 641.3 pg/mL; *p* = 0.0218) (Figure 2a). Similarly, for IL-8, significant differences were found between definite- and suspected-SBP (median: 679.5 pg/mL vs. 13.3 pg/mL; *p* = 0.0074), and between definite- and no-SBP (median: 679.5 pg/mL vs. 3.5 pg/mL; *p* = 0.0278) (Figure 2b). Additionally, IL-6 and IL-8 levels showed positive correlations with ascitic WBC count and bacterial counts detected by Tm mapping (Figure 2c–f).

### 3.5. Dynamics of Macrophages in Ascitic Fluid

Flow cytometric analysis was performed on ascitic fluid samples from 27 patients; however, two samples could not be analyzed due to insufficient fluid volume. In cases where the Tm mapping was positive, the percentage of M1 macrophages was increased relative to M2 macrophages, resulting in a higher M1/M2 ratio. Representative data (case 4, M1/M2 ratio: 2.95) are shown in Figure S1a. M1 macrophages were elevated in a patient with suspected SBP (0.56%, middle panel of Figure S1a), while M2 macrophages remained unchanged (0.19%, right panel of Figure S1a). Although not statistically significant, the M1/M2 ratio tended to be higher in the Tm mapping-positive group (mean: 5.39 vs. 3.35; *p* = 0.6071) (Figure S1b).

### 3.6. Contribution of EVs to Ascitic Inflammation in Clinical Settings

**Figure 3:**
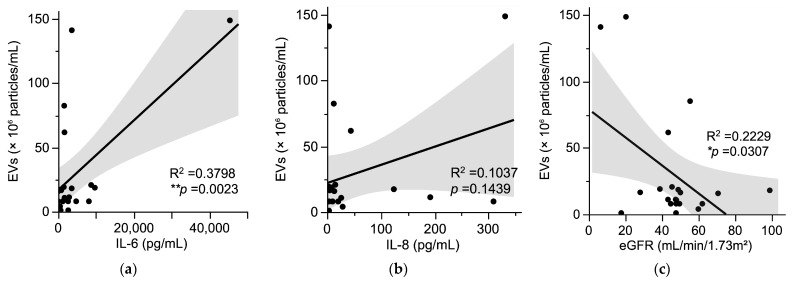
Analysis of EVs in ascitic fluid. (**a**,**b**) Correlation between EVs and cytokine levels in ascitic fluid measured by ELISA. (**a**) Correlation between EVs and IL-6 levels in ascitic fluid measured by ELISA. (**b**) Correlation between EVs and IL-8 levels in ascitic fluid measured by ELISA. (**c**) Correlation between EVs in ascitic fluid measured by ELISA and serum eGFR. The X-axis represents cytokine levels (IL-6 and IL-8) (pg/mL) measured by ELISA, as well as eGFR (mL/min/1.73 m^2^) values obtained from blood tests. The Y-axis represents EV levels (×10^6^ particles/mL) in ascitic fluid measured by ELISA. Scatter plots include regression lines with shaded areas indicating a 95% confidence interval. Regression analysis was performed to assess relationships between variables, with the coefficient of determination (R^2^) and *p*-values shown (* *p* < 0.05, ** *p* < 0.01).

To assess the contribution of specific PAMPs to ascitic inflammation, we examined the correlation between possible PAMPs and cytokine levels. LPS was detected in the ascitic fluid of 16 out of 26 cases in this cohort (Table 2), including all 3 cases of definite SBP, 9 out of 15 cases of suspected SBP, and 4 out of 8 cases of no SBP. We also used an ELISA kit to detect EVs derived from Gram-negative bacteria. EVs were detected in the ascitic fluid of 24 out of 26 cases in the cohort (Table 2). Among seven suspected SBP cases where Tm mapping was negative (Case 8 and Cases 15–20), EVs were detected in five out of six cases (one case was not evaluated). Although no statistically significant differences were observed, cases with Tm mapping-positive tended to exhibit higher levels of IL-6, IL-8, and EVs (Figure S2). There was no significant correlation observed between LPS levels and cytokines; a positive correlation was observed between IL-6 and EVs, but not between IL-8 and EVs (Figure 3a,b). Furthermore, the concentration of EVs in ascitic fluid was negatively correlated with estimated glomerular filtration rate (eGFR), a marker of renal function. Patients with impaired renal function tended to exhibit higher levels of EVs in ascitic fluid (Figure 3c).

### 3.7. Contribution of EVs to Macrophage-Induced Inflammation

**Figure 4:**
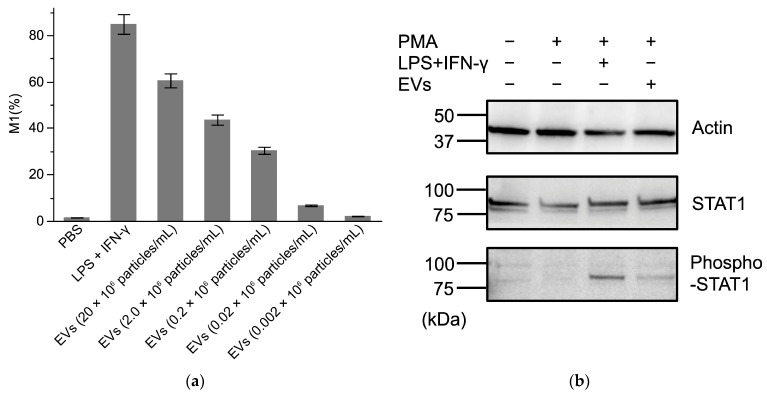
Differentiation of M1 macrophages induced by EVs from Gram-negative bacteria. (**a**) CD11b+CD80+ cells were classified as M1 macrophages, and the percentage of M1 macrophages relative to the total cell count was plotted on the Y-axis. EVs were diluted to the indicated concentrations in culture medium. (**b**) Western blot analysis of THP-1 cells under different conditions: untreated THP-1 cells, THP-1 cells treated with 5 ng/mL of phorbol 12-myristate 13-acetate (M0 macrophages), M0 macrophages stimulated with 10 ng/mL of LPS + 10 ng/mL of IFN-γ (M1 macrophages), and M0 macrophages treated with 1000-fold diluted EVs from Gram-negative bacteria. Activation pathways of each condition were analyzed. Representative data from three independent experiments are shown. PBS: phosphate-buffered saline, PMA: phorbol 12-myristate 13-acetate.

To analyze the mechanism of ascitic macrophage differentiation into M1 macrophages, we stimulated THP-1 cells with EVs derived from *E. coli* DH5α. Flow cytometric analysis demonstrated that EV stimulation induced differentiation into M1 macrophages in a dose-dependent manner (Figure S3 and Figure 4a). Furthermore, phosphorylation of STAT1 increased upon EV stimulation alone, similar to the response observed with LPS plus IFN-γ administration (Figure 4b).

## 4. Discussion

In this study, we performed Tm mapping using ascitic fluid samples from patients with decompensated cirrhosis and demonstrated its utility in diagnosing SBP. Although conventional cultures were negative in most cases, Tm mapping detected causative bacteria with greater sensitivity. In positive cases, elevated inflammatory cytokine levels in ascitic fluid were observed. Tm mapping enables earlier identification of causative bacteria in ascitic fluid, facilitating more appropriate treatment for SBP and potentially improving prognosis. To our knowledge, no studies have reported the utility of Tm mapping in ascitic fluid samples. Although originally developed for septic patients and validated in blood samples [11], Tm mapping has also been applied to other biological fluids, such as saliva and synovial fluid [13,14,15,16].

Recent studies have investigated the application of metagenomic analysis for the diagnosis of SBP [26]. Metagenomic sequencing enables the direct identification of causative pathogens based on DNA sequences with exceptionally high sensitivity. In contrast, Tm mapping offers several advantages, including relatively lower cost and a shorter turnaround time. Furthermore, Tm mapping can be performed using real-time PCR instruments, which are widely available and do not require the specialized equipment or computational infrastructure necessary for metagenomic analysis. Therefore, Tm mapping may be feasible for implementation across diverse clinical settings. Our findings suggest that Tm mapping could be a novel and effective method for SBP management, though further research is needed to confirm its efficacy.

Additionally, we investigated the relationship between ascitic inflammation and causative pathogens. We observed macrophage activation accompanied by increased production of inflammatory cytokines. IL-6 is rapidly secreted in response to infection or tissue injury, contributing to host defense by promoting acute-phase inflammation, while IL-8 promotes neutrophil recruitment, cell proliferation, and angiogenesis [27,28]. These cytokines, primarily secreted by macrophages, play a major role in initiating inflammatory responses. Previous studies have demonstrated that IL-6 and IL-8 levels are elevated in the ascitic fluid of SBP patients compared to those with sterile ascites, and blood IL-6 levels decrease following antibiotic treatment [29,30,31,32]. These findings suggest that SBP pathogenesis is strongly linked to these cytokines and that macrophages in ascitic fluid play a central role in disease progression. Consistently, our study also found that significantly higher levels of IL-6 and IL-8 in ascitic fluid from patients with confirmed SBP compared to those without. These cytokine levels were correlated with both the presence of bacteria and inflammatory findings in ascitic fluid. Notably, increases in ascitic WBC count or IL-6 were observed in some cases where Tm mapping was negative, suggesting that some pathogenic ligands may stimulate macrophages in ascitic fluid even before bacteria become detectable.

PAMPs activate immune cells such as macrophages and dendritic cells, inducing production of inflammatory cytokines [33]. LPS, a component of the outer membrane of Gram-negative bacteria, is one of the representative PAMPs [34]. LPS binds to toll-like receptor 4 (TLR4) on macrophages, triggering signaling pathways that activate transcription factor NF-κB [33,35]. This signaling promotes differentiation into M1 macrophages and stimulates the secretion of inflammatory cytokines such as IL-6 and IL-8 [34,36,37,38]. These findings suggest that LPS, by binding to TLR4, can activate macrophages and induce inflammation even in the absence of bacteria. Additionally, in patients with decompensated liver cirrhosis, LPS is thought to translocate into the liver, peritoneal cavity, and systemic circulation due to impaired gut barrier function and changes in gut microbiota, contributing to systemic inflammation [39]. Furthermore, LPS can also be transported by EVs—lipid bilayer-enclosed particles released by various cell types—that play roles in intercellular communication, immune dysfunction, and inflammation signaling [21,22,39,40,41]. Recent studies have shown the role of bacteria-derived EVs in various pathological processes [21,39,40,41,42,43]. Although the precise function of EVs remains unclear, studies have suggested they play a role in liver disease. One study demonstrated that EVs induce hepatic inflammation in a mouse model of liver cirrhosis through macrophage activation and detected bacteria-derived EVs in the ascitic fluid of patients with decompensated cirrhosis but not in those with pancreatic cancer [41]. These findings suggest that bacteria-derived EVs may contribute to cirrhosis-related inflammation in both experimental and clinical settings. Similarly, in our study, EVs were detected in the ascitic fluid of most cases, and a positive correlation was observed between EV levels and IL-6 concentrations. In addition, Tm mapping-positive cases tended to exhibit elevated levels of EVs and the cytokines IL-6 and IL-8. Tm mapping is a diagnostic method capable of detecting minute amounts of bacteria that cannot be identified by conventional culture methods. Although the small sample size in this study limited the ability to detect statistically significant differences, these findings suggest that such low-level bacteria, or the EVs released from them, may activate macrophages within the peritoneal cavity, promoting the release of cytokines such as IL-6 and IL-8 and contributing to early immune activation. Furthermore, in vitro stimulation with EVs alone was sufficient to induce M1 macrophage differentiation. These findings suggest that even in the absence of bacteria, the presence of EVs can induce an inflammatory response in ascitic fluid.

Several signaling pathways contribute to the differentiation of macrophages into the M1 phenotype [44,45,46]. Among these, the interferon regulatory factor/signal transducer and activator of transcription (IRF/STAT) signaling pathway is activated by IFN and TLR signaling, promoting M1 macrophage differentiation via STAT1. Additionally, the LPS/TLR4 pathway can activate STAT-α/β through a MyD88-independent mechanism. It is well established that combined stimulation with LPS and IFN-γ classically induces macrophage differentiation in the M1 phenotype [47]. Other studies have also reported that LPS + IFN-γ induces stronger expression of M1-associated genes than LPS alone [48,49]. In our study, no significant correlation was found between ascitic LPS levels and IL-6 concentrations. However, stimulation with EVs alone activated phosphorylated STAT1, similar to the effect observed with combined LPS and IFN-γ stimulation. During M1 macrophage differentiation, STAT1 is activated by IFN-γ and promotes polarization into M1 macrophages [48,49]. These findings suggest that, in addition to LPS, certain proteins within EVs may induce IFN-γ signaling. However, the contribution of exosomes could not be evaluated in this study. Although the precise signaling pathway remains unclear, our findings indicate that EVs alone can induce M1 macrophage differentiation, suggesting their potential role in triggering inflammation in ascitic fluid. Recent studies have reported that gut-derived EVs can propagate inflammation signals to the liver and systemic circulation via the gut–liver axis [41]. The presence of EVs in ascitic fluid and their macrophage-activating effects observed in this study may be closely associated with disruptions of the gut–liver axis, such as alterations in the gut microbiota and impaired intestinal barrier function. Further investigation is needed to clarify the origin of EVs in ascitic fluid and their pathological significance.

In this study, a correlation was observed between EVs in ascitic fluid and renal function, as indicated by eGFR. Patients with impaired renal function tended to exhibit higher levels of EVs in ascitic fluid. Renal dysfunction has been closely associated with poor prognosis in patients with liver cirrhosis [50]. Although no direct correlation was found between EV levels and the MELD-Na score or serum bilirubin (), the findings suggest a potential association between increased EV levels in ascitic fluid and worse clinical outcomes in cirrhosis. Furthermore, previous studies have reported that patients with renal dysfunction often exhibit compromised intestinal barrier function and increased intestinal permeability [51]. This “leaky gut” condition may contribute to the elevated levels of EVs detected in ascitic fluid. However, the underlying mechanisms remain unclear and warrant further investigation.

This study has several limitations. First, the small sample size limited comparisons of patient backgrounds and clinical profiles. Additionally, the hepatic venous pressure gradient was not measured, making a definitive diagnosis of portal hypertension difficult. Although conducting clinical studies on decompensated patients with cirrhosis with detailed background data is challenging, further studies with larger populations are needed. Second, Tm mapping is unable to identify specific bacterial species in polymicrobial infections. Nevertheless, it offers superior sensitivity in detecting the presence of bacteria compared to conventional culture methods. Therefore, combining Tm mapping with conventional ascitic fluid cultures may allow for earlier and more accurate intervention in SBP. Third, flow cytometric analysis of ascitic fluid was limited by low cellularity and contamination with mesothelial cells, which resulted in high background noise and reduced data clarity. Furthermore, the relationship between causative bacteria/EVs in ascitic fluid and the gut microbiota could not be evaluated, as stool samples were not collected for microbial analysis. In some cases, even when ascitic fluid cultures were positive, EV concentrations in ascitic fluid were sometimes low, and the number of bacterial cells detected by Tm mapping did not always correlate with EV levels. The precise mechanisms by which EVs are transferred into ascitic fluid remain poorly understood. Finally, the EVs used in the in vitro experiments were derived from *E. coli*, whereas ascitic fluid likely contains various bacterial species, which may lead to differences in clinical findings. Further studies are needed to clarify the role of EVs in ascitic inflammation.

## 5. Conclusions

We demonstrated the utility of the Tm mapping method for diagnosing SBP and confirmed the presence of EVs derived from Gram-negative bacteria in the ascitic fluid of patients with cirrhosis. The presence of EVs may be associated with the prognosis of cirrhosis by triggering ascitic macrophage-induced inflammation. While many aspects of EV function remain unclear, further research is needed to elucidate their role in disease progression.

## Figures and Tables

**Table 1:** Background characteristics of all patients.

	*n* = 29 Numbers or Median (Range)
Age (years)	69 (48–94)
Gender (male/female)	17/12
Etiology (HBV/HCV/MASH/Alc/others)	2/3/8/9/7
Child–Pugh score	10 (8–14)
Grade A/B/C	0/8/21
MELD-Na score	10.1 (0.8–23.8)
Fib-4 index	6.83 (1.46–41.51)
Antibiotic therapy ^1^	10
Complications	
Varices (absent/present)	17/12
HCC (absent/present)	21/8
Hepatic encephalopathy (absent/present)	22/7
Symptoms	
Fever (absent/present)	14/15
Abdominal pain (absent/present)	24/5
Diarrhea (absent/present)	23/6
Nausea/Vomiting (absent/present)	27/2
Laboratory data	
WBC (/μL)	5940 (2640–21,900)
CRP (mg/dL)	1.58 (0.14–18.9)
Platelets (×10^4^ /μL)	8.5 (1.5–22.6)
Albumin (g/dL)	2.2 (1.0–3.2)
Creatinine (mg/dL)	1.08 (0.57–8.18)
Ascites paracentesis	
WBC (/μL)	330 (30–13740)
Albumin (g/dL)	0.6 (0.1–1.8)
SAAG	1.6 (0.8–2.4)

HBV, hepatitis B virus; HCV, hepatitis C virus; MASH, metabolic dysfunction-associated steatohepatitis; Alc, alcohol; HCC, hepatocellular carcinoma; MELD, model for end-stage liver disease; WBC, white blood cell; CRP, C-reactive protein; SAAG, serum ascites albumin gradient. ^1^ At the time of ascites puncture.

**Table 2:** Ascites bacterial test results using the Tm mapping method, conventional method, and ELISA analysis of PAMPs.

Case	Clinical Presentation	Conventional Methods		Tm Mapping Method		Results of ELISA	
		Bacterial Name	Ascites-WBC (/μL)	Bacterial Name	Bacterial Concentrations (/mL)	LPS (ng/mL)	EVs (×10^6^ Particles/mL)
1	Definite SBP	Enterococcus.faecium	9800	Enterococcus.faecium	95,433	0.05	3.24
2	Definite SBP	Klebsiella.pneumoniae	13,740	Klebsiella.pneumoniae	2707	0.09	11.21
3	Definite SBP	Klebsiella.pneumoniae	5040	Multiple	19,900	0.07	148.87
4	Suspected SBP	ND	940	Eggerthella lenta	199	0.12	6.61
5	Suspected SBP	ND	1860	Streptococcus mitis	56	NE	NE
6	Suspected SBP	ND	430	Bacillus cereus	12.9	<0.01	16.60
7	Suspected SBP	ND	920	Multiple	50	0.09	11.21
8	Suspected SBP	ND	410	ND	ND	<0.01	1.11
9	Suspected SBP	ND	710	Unknown	50	0.05	15.84
10	Suspected SBP	ND	440	Unknown	25	0.07	10.76
11	Suspected SBP	ND	90	Unknown	19	<0.01	4.79
12	Suspected SBP	ND	2120	Unknown	6	0.04	252.54
13	Suspected SBP	ND	370	Unknown	6	0.03	141.22
14	Suspected SBP	ND	490	Unknown	2	<0.01	82.43
15	Suspected SBP	ND	310	ND	ND	NE	NE
16	Suspected SBP	ND	140	ND	ND	0.02	18.63
17	Suspected SBP	ND	30	ND	ND	<0.01	6.08
18	Suspected SBP	ND	180	ND	ND	<0.01	20.72
19	Suspected SBP	ND	1500	ND	ND	0.07	19.13
20	Suspected SBP	ND	120	ND	ND	0.02	<1.00
21	No SBP	ND	260	ND	ND	NE	NE
22	No SBP	ND	250	ND	ND	<0.01	61.73
23	No SBP	ND	120	Multiple	25	0.47	17.3
24	No SBP	ND	150	ND	ND	<0.01	16.47
25	No SBP	ND	200	ND	ND	0.01	18.09
26	No SBP	ND	330	ND	ND	<0.01	<1.00
27	No SBP	ND	140	ND	ND	<0.01	4.39
28	No SBP	ND	130	ND	ND	0.12	6.61
29	No SBP	ND	320	ND	ND	0.18	85.32

For all patients, the clinical features, results of Tm mapping, results of conventional diagnostic tests, and results of ELISA analysis of PAMPs are shown. Bacterial name indicates the identified bacterial species, but if multiple bacteria were detected, it was recorded as “Multiple,” and if the bacterial species could not be identified, it was recorded as “Unknown.” Ascites-WBC: ascitic WBC (white blood cell) count, NE: not evaluated, ND: not detected.

## Data Availability

The data shown in this study are available from the corresponding author upon reasonable request.

## Supplementary Materials

The following supporting information can be downloaded at https://www.mdpi.com/article/10.3390/jcm14145096/s1, Figure S1: Dynamics of macrophages in ascitic fluid. (a) Flow cytometry analysis of ascitic fluid from a patient (representative data from Case 4). CD11b+CD80+ cells were classified as M1 macrophages, while CD11b+CD163+ cells were classified as M2 macrophages. The percentage of each cell number is shown in the graph. (b) Correlation between Tm mapping results and macrophage dynamics. The M1/M2 ratio was calculated from the percentage of cells differentiated into M1 and the percentage of cells differentiated into M2, and the average value (bar graph) and 95% confidence interval for each group are shown. Tm mapping was defined as positive when bacteria were detected by the Tm mapping method. Figure S2: Correlation between Tm mapping results and cytokine/EVs levels in ascitic fluid. (a) Correlation between Tm mapping results and IL-6 levels in ascitic fluid measured by ELISA. (b) Correlation between Tm mapping results and IL-8 levels in ascitic fluid measured by ELISA. (c) Correlation between Tm mapping results and EV levels in ascitic fluid measured by ELISA. The average value (bar graph) and 95% confidence interval for each group are shown. Tm mapping was defined as positive when bacteria were detected by the Tm mapping method. Figure S3: Examination of differentiation conditions for THP-1 cells. THP-1 cells were treated with 5 ng/mL of phorbol 12-myristate 13-acetate for 24 h, washed three times with PBS, and incubated with fresh medium for 72 h before stimulation with the indicated concentrations of LPS + IFN-γ and EVs. CD11b+/CD80+ cells were classified as M1 macrophages and analyzed by flow cytometry. The percentages of CD11b+/CD80+, CD11b-/CD80+, CD11b+/CD80-, and CD11b-/CD80- cells are shown in the graph.

## Abbreviations

The following abbreviations are used in this manuscript:

SBP	Spontaneous bacterial peritonitis
Tm	Melting temperature
E. coli	Escherichia coli
PAMPs	Pathogen-associated molecular pattern molecules
EVs	Extracellular vesicles
LPS	Lipopolysaccharide
WBC	White blood cell
DV	Difference value
IFN	Interferon
ELISA	Enzyme-linked immunosorbent assay
IL	Interleukin
MELD-Na score	Model for End-Stage Liver Disease Sodium score
SI	Splenic index
TLR4	Toll-like receptor 4
eGFR	Estimated glomerular filtration rate
STAT1	Signal transducer and activator of transcription
IRF/STAT	Interferon regulatory factor/signal transducer and activator of transcription
