# Supplementary material for: Spontaneous Bacterial Peritonitis in Advanced Cirrhosis: Diagnosis by Tm Mapping and Inflammatory Profiles of Extracellular Vesicles
Source: J Clin Med. 2025 Jul 17;14(14):5096. doi: 10.3390/jcm14145096 (PMC12295209; doi:10.3390/jcm14145096)
Supplement: Supplementary file 1 [file jcm-14-05096-s001.zip › jcm-3707232-supplementary.pdf]

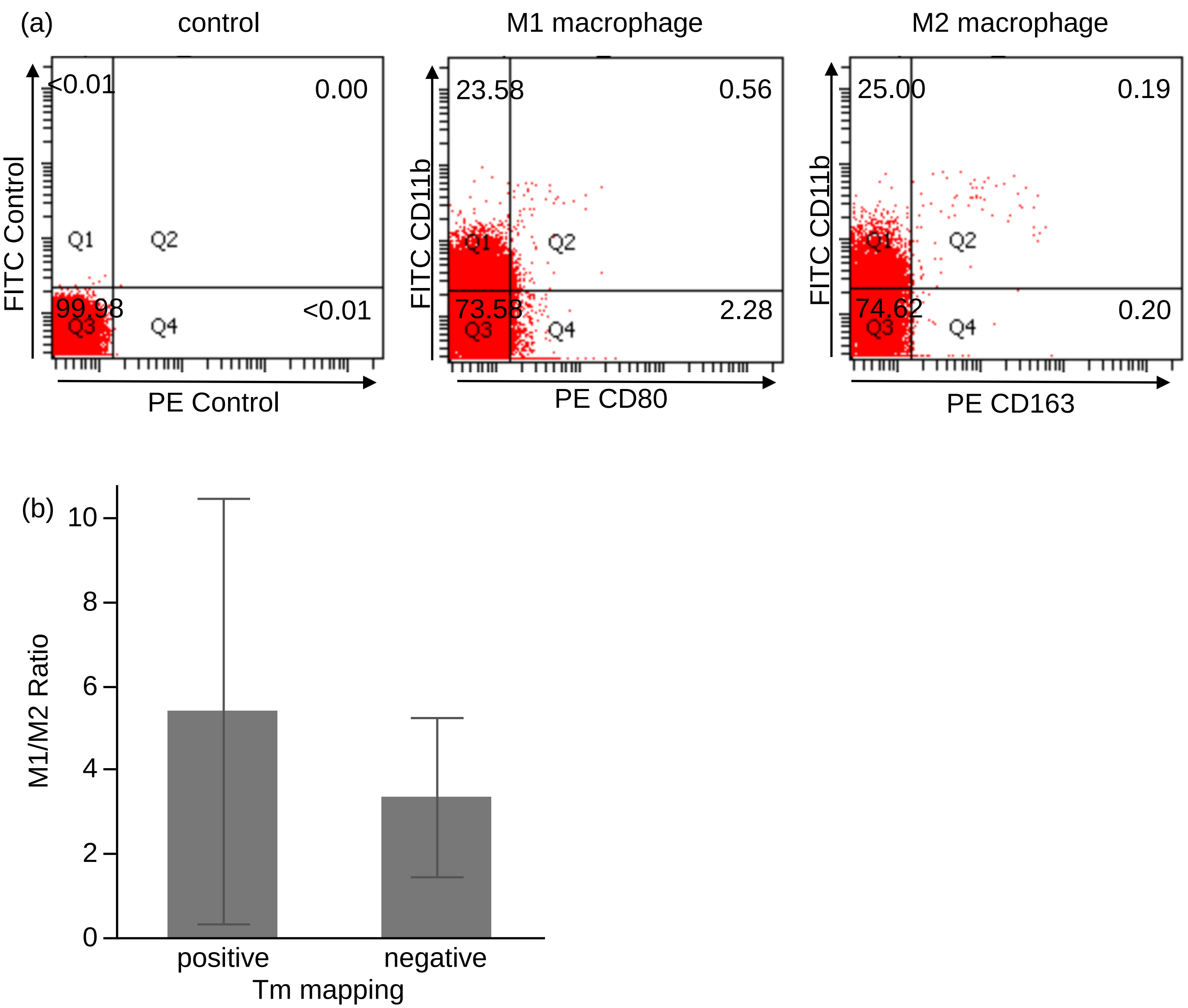

Figure S1: Dynamics of macrophages in ascitic fluid. (a) Flow cytometry analysis of ascitic fluid from a patient (representative data from Case 4). CD11b+CD80+ cells were classified as M1 macrophages, while CD11b+CD163+ cells were classified as M2 macrophages. The percentage of each cell number is shown in the graph. (b) Correlation between Tm mapping results and macrophage dynamics. The M1/M2 ratio was calculated from the percentage of cells differentiated into M1 and the percentage of cells differentiated into M2, and the average value (bar graph) and 95% confidence interval for each group are shown. Tm mapping was defined as positive when bacteria were detected by the Tm mapping method.

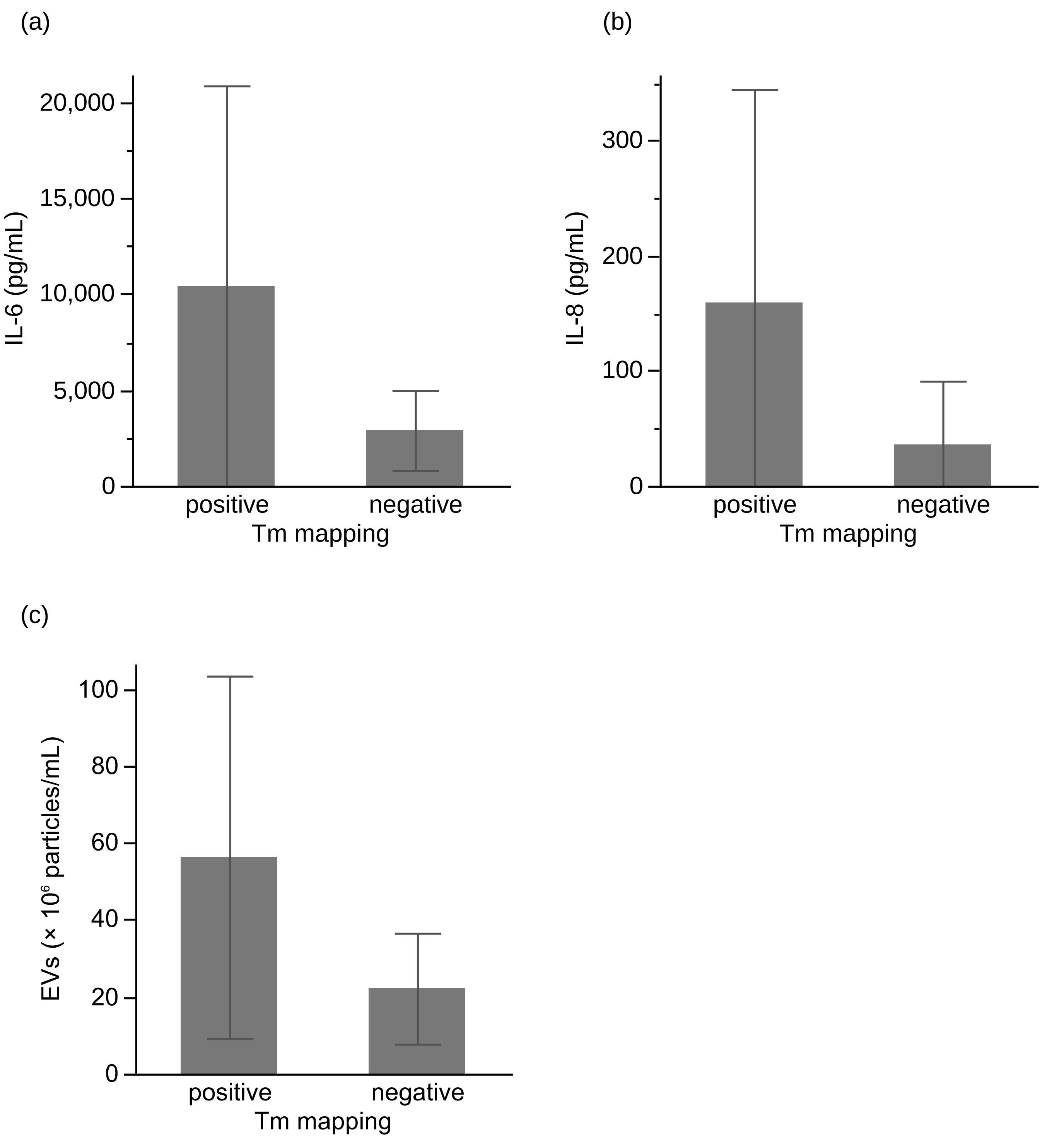

Fig S2. Correlation between Tm mapping results and cytokine/EVs levels in ascitic fluid.

(a) Correlation between Tm mapping results and IL-6 levels in ascitic fluid measured by ELISA. (b) Correlation between Tm mapping results and IL-8 levels in ascitic fluid measured by ELISA. (c) Correlation between Tm mapping results and EVs levels in ascitic fluid measured by ELISA. The average value (bar graph) and 95% confidence interval for each group are shown. Tm mapping was defined as positive when bacteria were detected by the Tm mapping method.

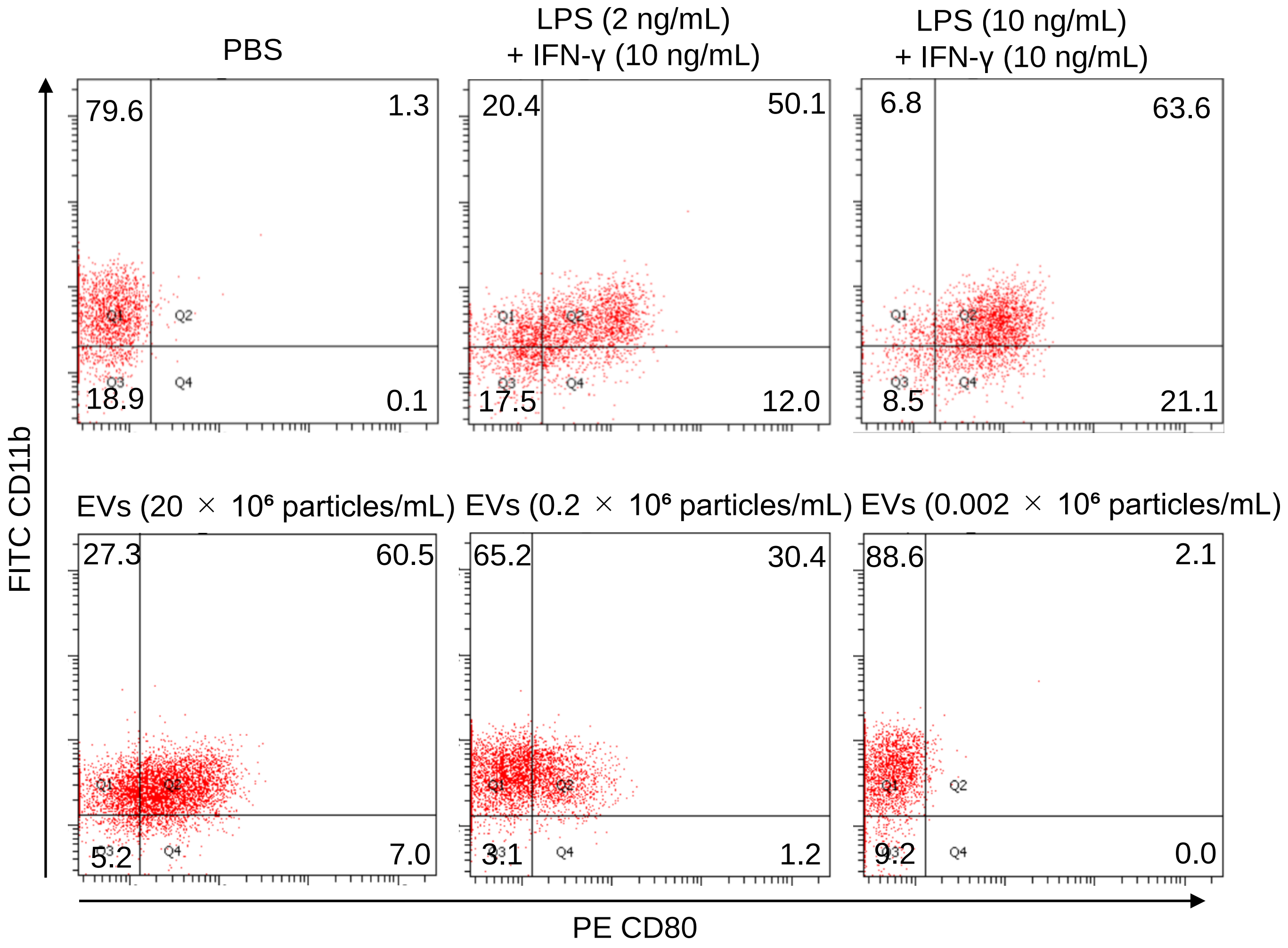

Fig S3. Examination of differentiation conditions for THP-1 cells

THP-1 cells were treated with 5 ng/mL phorbol 12-myristate 13-acetate for 24 hours, washed three times with PBS, and incubated with fresh medium for 72 hours before stimulation with the indicated concentrations of LPS + IFN-γ and EVs. CD11b+/CD80+ cells were classified as M1 macrophages and analyzed by flow cytometry. The percentages of CD11b+/CD80+, CD11b-/CD80+, CD11b+/CD80-, and CD11b-/CD80- cells are shown in the graph.
